# Influence of Si Addition on the Microstructures, Phase Assemblages and Properties in CoCrNi Medium-Entropy Alloy

**DOI:** 10.3390/ma17122893

**Published:** 2024-06-13

**Authors:** Hui Chang, Shengfang Wang, Zhouzhu Mao, Tuanwei Zhang, Zhiqiang Li, Zhihua Wang

**Affiliations:** 1 Institute of Applied Mechanics, College of Mechanical and Vehicle Engineering, Taiyuan University of Technology, Taiyuan 030024, China; cchanghui001@163.com (H.C.);; 2 Shanxi Key Laboratory of Material Strength and Structural Impact, Taiyuan University of Technology, Taiyuan 030024, China; 3 College of Aeronautics and Astronautics, Taiyuan University of Technology, Taiyuan 030024, China

**Keywords:** medium-entropy alloys, mechanical properties, microstructures, hardness, wear resistance

## Abstract

The effects of Si addition on the microstructures and properties of CoCrNi medium-entropy alloy (MEA) were systematically investigated. The CrCoNiSi_x_ MEA possesses a single face-centered cubic (FCC) phase when x is less than 0.3 and promotes solution strengthening, while the crystal structure shows a transition to the FCC+σ phase structure when x = 0.4 and the volume fraction of the σ phase increases with a microstructure evolution as the Si content increases. The Orowan mechanism from σ precipitation effectively enhances the strength, hardness, and stain hardening of CrCoNiSi_x_ MEA, which also exhibits superior hardness at high temperatures. Furthermore, a large amount of σ phase decreases the wear resistance because of the transformation of the main wear mechanism from abrasion wear for σ-free CrCoNiSi_x_ MEA to adhesion wear for σ-contained CrCoNiSi_x_ MEA. This work contributes to the understanding of the effect of Si addition on FCC structured alloys and provides guidance for the development of novel Si-doped alloys.

## 1. Introduction

In the last two decades, medium- and high-entropy alloys (M/HEAs) have been widely developed and studied due to their unique structures and superior comprehensive mechanical properties to traditional alloys [1,2,3]. It is reasonable to assume that promising properties in hardness, strength, wear resistance, corrosion resistance and oxidation resistance can be realized by composition design [4,5,6]. It is known that the proper addition of elements can change the microstructure and optimize the properties of alloys [5,6,7,8]. In particular, the non-metallic elements, such as Si, C, and B, may promote solution strengthening due to large lattice distortion or precipitation strengthening due to the introduction of intermetallic material with Si, C, or B to realize a constructive role in tailoring the microstructure and properties [8,9,10]. Hence, it is significant to study the effect of the non-metallic element addition on the microstructure and properties of the alloys.

Many researchers have studied and proved that the addition of Si element significantly alters the phase assemblages and enhances the alloy’s hardness, wear resistance, corrosion resistance, and oxidation resistance [11,12,13,14,15,16,17,18,19,20,21,22]. The FeCoNiCrSi_x_ [11], Al_0.5_CoCrCuFeNiSi_x_ [12], FeCoNiAlSi_x_ [13], Al_0.3_CoCrFeNi [14], Fe_2.5_CoNiCu [15], and AlCoCuNi-based M/HEAs [16] undergo the transition from a closed-packed face-centered cubic (FCC) structure to a loose-packed body-centered cubic (BCC) structure with the increasing of Si content, thus leading to better hardness and wear resistance. As Si content increases, the, FeCoCrNiMoSi_x_ [17], Co_0.2_CrAlNi [18], FeCrMnVSi_x_ [19], and CoCrFeNiSi_x_ [20] M/HEAs enhance the hardness and wear resistance through the introduction of Si-rich precipitation, such as Cr_3_Si, Si-Ni, and σ phase. The CoCrCuFeNiSi_x_ [21] and FeCoCrNiAl_0.5_Si_x_ [22] HEAs were reported to enhance the strength, hardness, and wear resistance by the transition from FCC to BCC and the formation of an intermetallic precipitation Cr_3_Si phase. Therefore, the addition and increase in the Si element can effectively change the microstructure of alloys and thus enhance the properties which are required for studying.

In our previous study, the Si addition in CrCoNi MEA promotes single-phase FCC solid solution strengthening when x ≤ 0.3 [8]. Meanwhile, it reduces the stacking fault energy and increases the short-range order, thus effectively improving the work hardening ability of the alloy and achieving the synergistical enhancement in strength and plasticity [8]. In previous work, we aimed to improve the combination of strength and ductility in the range of keeping the single FCC phase. Nevertheless, the increase in Si content usually influences the crystal structure to change the mechanical performance [11,12,13,14,15,16,17,18,19,20,21,22]. Therefore, now we focus on the transformation of the crystal structure and mechanical properties with increasing the Si content. How the microstructure and mechanical properties of the alloy vary with more Si content? Will it introduce the appearance of other Si-rich precipitated phases? The systematic study of Si element on the microstructure and properties of CrCoNiSi_x_ MEA is required in order to answer these questions. Furthermore, the Si element generally increases the wear properties of the alloy [11,12,13,14,15,16,17,18,19,20,21,22]; nevertheless, the wear properties and mechanisms of CrCoNiSi_x_ MEA have not been studied.

In the present paper, the effects of Si content on the microstructures and phase assemblages of CrCoNiSi_x_ MEA are systematically investigated. The corresponding mechanical and wear properties are also studied. The increase in the Si element leads to the transition of the alloy phase structure and further promotes the improvement of alloy properties. The strengthening mechanisms of the alloy are discussed in detail.

## 2. Material and Experimental Methods

### 2.1. Material Preparation

Alloy ingots with nominal compositions of CrCoNiSi_x_ (x values in molar ratio, x = 0, 0.1, 0.2, 0.3, 0.4, 0.5, 0.6, denoted by Si_0_, Si_0.1_, Si_0.2_, Si_0.3_, Si_0.4_, Si_0.5_ and Si_0.6_, respectively) were prepared by arc-melting the mixtures of metals (purity > 99.9 wt.%) in a high-purity argon atmosphere. The ingots were flipped and remelted five times to ensure chemical homogeneity, followed by drop-casting in a water-cooled copper mold with dimensions of 2 × 10 × 100 mm^3^. The casted plates were homogenized at 1100 °C for five hours followed by water quenching, which is in accordance with the previous study [8] for comparison. In an attempt to roll the homogenized specimens, the CrCoNiSi_x_ (x = 0.4, 0.5, 0.6) MEAs crack when rolled to 35% thickness reduction and cannot be rolled to 70% thickness reduction, as seen in [8], so the homogenized specimens for all MEAs were obtained and used for study in present paper.

### 2.2. Mechanical Experiment

Dog-bone-shaped tensile specimens with a gage geometry (length × width × thickness) of 10.0 × 4.0 × 2 mm^3^ were cut for quasi-static tension samples, and the cylinder specimens with a diameter of ϕ 3 mm and a height of 3 mm were adopted for compression tests. The tensile and compression tests experiments were performed using an Instron 5969 (Instron, a Division of Illinois Tool Works Inc. ITW, Norwood, MA, USA) machine at a constant strain rate of 1 × 10^−3^ s^−1^. Vickers hardness was measured using a THV-1DTe tester with a load of 0.5 N and a duration time of 10 s for each measurement. The hardness values were averaged by at least ten measurements for each sample.

Wear testing was performed on a reciprocating friction testing machine (CETR-UMT 2MT, Labs Arena, San Clemente, CA, USA) using a 6 mm diameter GCr15 steel ball as the contact counterpart at room temperature. The tests were conducted at the load of 20 N under a constant velocity of 6 mm/s and a stroke length of 3 mm for a total duration of 30 min. The wear tests were repeated three times with a new steel ball and the wear weights of the specimens were averaged using an analytical balance. The microstructures of the worn surface were characterized using scanning electron microscopy (SEM).

### 2.3. Microstructural Characterization

The X-ray diffraction (XRD) measurements were performed using the Rigaku Ultima IV diffractometer under Cu-Kα radiation at 40 kV and 40 mA (scanning rate = 1°/min, 2θ = 40–100°, step = 0.01°). The microstructural characterizations were performed by JEOL JSM-7100F field emission gun-scanning electron microscopy (SEM) (JEOL, Peabody, MA, USA), back-scattered electrons (BSEs), dual energy-dispersive X-ray spectroscopy (Dual EDS) detectors, and an electron backscatter diffraction detector (EBSD) system at an acceleration voltage of 20 kV. More refined microstructures were obtained by a transmission electron microscope (TEM; JEM-2100F, JEOL, Peabody, MA, USA) at an acceleration voltage of 200 kV. TEM specimens were prepared by mechanical grinding down to 30 µm thickness and then thinned using double-jet electropolishing in a solution of 95% ethanol and 5% perchloric acid at −20 °C and an applied voltage of 30 V, followed by Ar-ion milling.

## 3. Results and Discussion

### 3.1. Microstructures and Phase Assemblages

**Figure 1:**
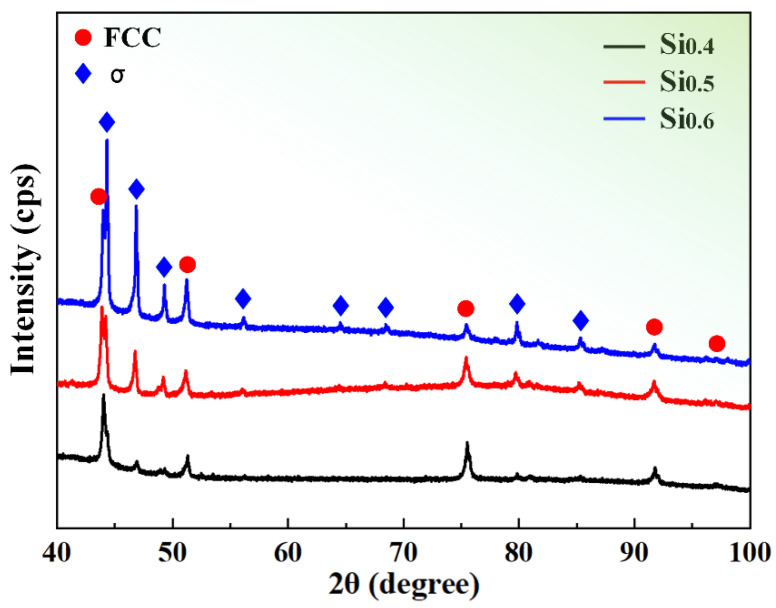
XRD patterns of the homogenized CrCoNiSi_x_ (x = 0.4, 0.5, 0.6) MEAs.

The crystal structures of recrystallized CrCoNiSi_x_ (x = 0.4, 0.5, 0.6) MEAs were characterized by XRD patterns, as displayed in Figure 1. Except for the FCC phase, there were some other peaks corresponding to a tetragonal structure with the lattice parameters of a ≈ 8.8 Å and c ≈ 4.5 Å, which was indexed to the σ phase. As Si concentration increased, the peaks corresponding to the σ phase intensified, indicating the larger σ phase content. At x = 0, 0.1, 0.2, and 0.3, only the FCC-type solid solution structure was detected [8]. When x increased to 0.4, the phase assemblages became FCC+σ and the amount of σ phase was gradually enlarged with the Si increase.

**Figure 2:**
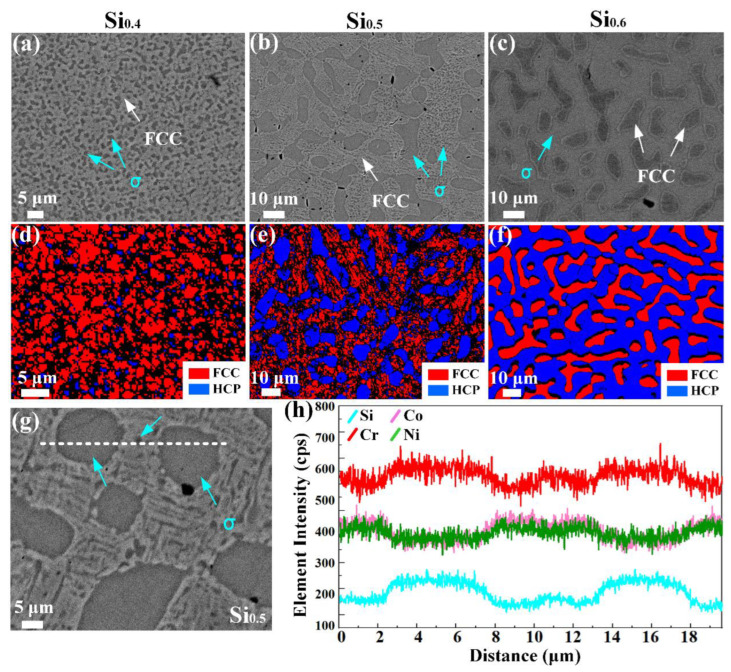
The back-scattered SEM images (**a**–**c**), EBSD phase maps (**d**–**f**) of homogenized CrCoNiSi_x_ (x = 0.4, 0.5, 0.6) MEAs, respectively. (**g**) Enlarged image of (**b**), (**h**) EDS line scan results of cross-section of the matrix and σ particles interfaces as white dotted line marked in (**h**) showing the variations in the compositional elements.

Figure 2 presents the BSE images and EBSD phase maps of recrystallized CrCoNiSi_x_ (x = 0.4, 0.5, 0.6) MEAs. It can be found that all three MEAs are composed of FCC and σ phases. Pay attention to the phase distribution of three MEAs to analyze the evolution of microstructures as the Si increases. The small σ-phase particles are uniformly distributed in the FCC matrix in CrCoNiSi_0.4_ MEA. In CrCoNiSi_0.5_ MEA, some parts of the small σ-phase particles grow and converge into large particles (with an average size of 10 μm), forming the large- and small-particle distributed network structure. When x reaches 0.6, all the small σ-phase particles grow and converge into a large-area σ phase and the volume fraction exceeds the FCC structure, showing that the FCC phase is distributed in the σ-phase structure. Figure 2h exhibits the proximity histograms of the elemental concentrations across the interface between the matrix and precipitates as white lines, as shown in Figure 2g. The line scan spans two large-sized and one small-sized σ particles. It can be clearly seen that the concentration of Si and Cr elements increases when crossing the interface from the FCC matrix to the σ phase. Cr and Si elements in the σ-precipitated phase are obviously larger than those in the FCC phase, indicating that Cr and Si elements are enriched in the σ phase.

**Figure 3:**
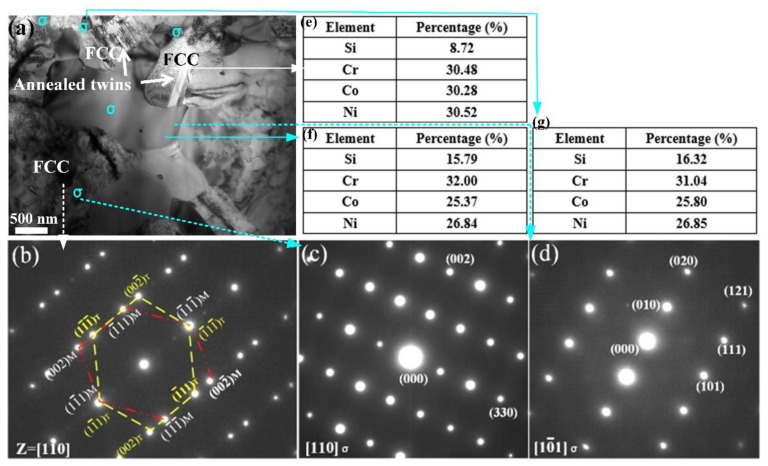
(**a**) Bright-field TEM image of the homogenized CrCoNiSi_0.5_ MEA; TEM-energy dispersive spectrometer (EDS) results reflexing the element composition in FCC matrix (**e**) and large and small particles (**f**,**g**). The corresponding SAED patterns for FCC matrix (**b**) and σ phase (**c**,**d**).

To further confirm the phase assemblages and element composition, the CrCoNiSi_0.5_ MEA with both large and small sized precipitates was selected for observation using transmission electron microscopy (TEM) technology. As shown in the bright-field image in Figure 3a, there are obvious annealing twins in the FCC matrix region, and the large and small particles (with an average size of 100 nm) are distributed in the FCC phase. The corresponding selected area electron diffraction (SAED) patterns exhibited in Figure 3b–d indicate the FCC structure with annealing twin and the σ-phase precipitation. This agrees with the results of XRD, BSE, and EBSD. In detailed element compositions of the FCC region, small and large σ-phase particles were recognized through TEM-EDS; the results are shown in Figure 3e–g. It was found that the proportion of elements in the FCC region was close to the molar ratio of the CrCoNiSi_0.5_ MEA, while the proportions of Si and Cr elements were relatively low, which is a result of Si, Cr element segregation in the σ phase. Additionally, the element proportion of the small particles is close to the large ones, and both of them are enriched with Si and Cr elements. This further proves the Si, Cr-rich σ phase and indicates that large and small σ-phase precipitates have the same element composition.

In summary, CrCoNiSi_x_ shows the transition from the pure FCC solid solution phase to the mixture of FCC and σ precipitation as the Si content increases. When x is less than 0.3, the MEA maintains an FCC single-phase structure, showing solid solution strengthening with Si increasing [8]. An apparent transition in phase assemblages occurs at x = 0.4 in which σ phase appears. As the molar ratio of Si increases, the σ phase gradually increases, showing the transition from a uniform distribution of small-sized precipitates to a large- and small-particle distributed network structure, and eventually to the FCC phase being distributed in the σ-phase matrix structure. In conclusion, small Si addition does not change the phase assemblages, but a higher content of Si tends to promote the formation of σ precipitation in CrCoNi MEA.

### 3.2. Mechanical Properties and Strengthening Mechanisms

**Figure 4:**
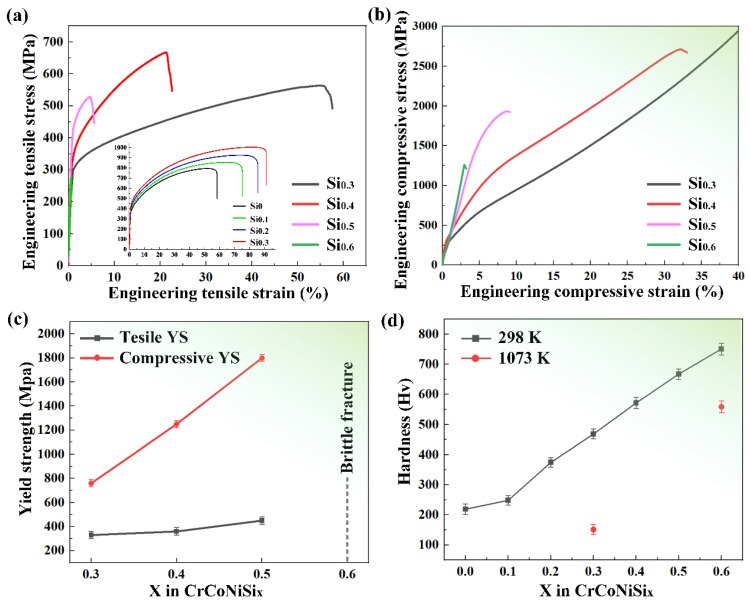
Quasi-static (**a**) tensile engineering stress–strain curves with the engineering strain−stress curves of the recrystallized CrCoNiSi_x_ (x = 0.3, 0.4, 0.5, 0.6) MEAs inserted [8] and (**b**) compressive engineering stress–strain curves of the homogenized CrCoNiSi_x_ (x = 0.3, 0.4, 0.5, 0.6) MEAs; (**c**) variations of tensile and compressive YS in CrCoNiSi_x_ with Si content; (**d**) variations in hardness of CrCoNiSi_x_ MEAs at room and high temperatures.

Figure 4a,b show the quasi-static tensile and compressive stress–strain curves of the homogenized Si_x_ (x = 0.3, 0.4, 0.5 and 0.6) MEAs, respectively, with the variations in tensile and compressive yield strength (YS) with Si content being displayed in Figure 4c. It can be seen that as the Si mole ratio increases from 0.3 to 0.5, the tensile and compressive yield strength increases from 320 to 430 MPa and from 760 to 1820 MPa, respectively, with a gradual decrease in plastic strain, while Si_0.6_ MEA shows a brittle fracture manner without yield strain. It is noticeable that the Si_0.4_ and Si_0.5_ MEAs also exhibit an obviously higher strain-hardening capacity than Si_0.3_ MEA, which can be attributed to the precipitate hardening of the σ phase. However, due to smaller plastic strain, the ultimate tensile and compressive strengths of Si_0.5_ MEA is less than Si_0.4_ MEA. In conclusion, small Si addition in CrCoNi MEA promotes solution strengthening and reduces the stacking-fault energy, thus realizing synergistical enhancement in strength and plasticity [8], and more Si content leads to precipitation strengthening and improves the strength and strain hardening effectively, but the large number of the σ phase causes a brittle fracture when Si content increases too much, which is not suitable for engineering structures.

The hardness variations in Si_x_ MEA are shown in Figure 4d. The hardness of Si_x_ MEA exhibits a continuous increase with Si content, and the Si_0.6_ MEA shows a hardness of 751.21 Hv, 282.93 Hv (increased by 60.5%) higher than Si_0.3_ MEA with a single FCC phase and 532.07 Hv (increased by 242.8%) higher than CrCoNi MEA, indicating that Si addition effectively enhances the hardness of the CrCoNi MEA, especially for the σ-phase-contained Si_x_ MEA. Moreover, the hardness of Si_0.3_ and Si_0.6_ MEAs at a high temperature (1073 K) are tested and the values are 151.2 Hv and 558.9 Hv, respectively. It can be observed that the hardness of Si_0.6_ MEA is increased by 269.6% compared to that of Si_0.3_ MEA. The increase percentage is much greater than that at room temperature. The hardness of Si_0.3_ and Si_0.6_ MEAs at high temperature is 317.08 Hv (decreased by 85%) and 192.31 Hv (decreased by 26%) lower than those at room temperature, respectively. This indicates that the σ phase effectively enhances the hardness of the CrCoNi MEA at high temperatures.

**Figure 5:**
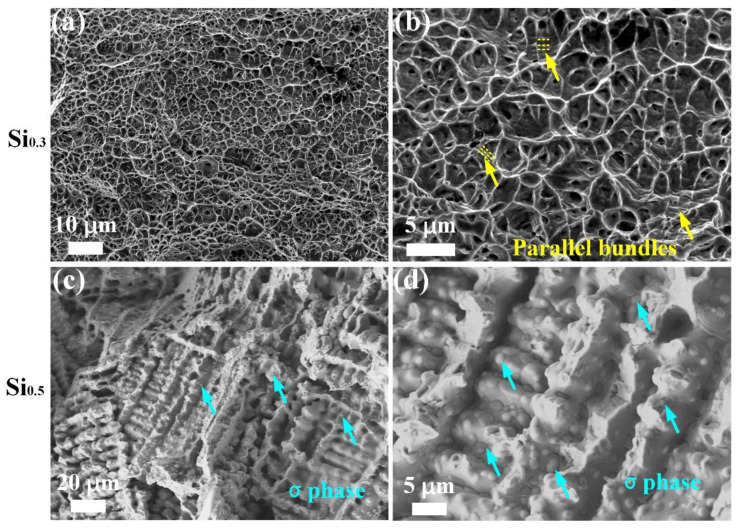
Fractographic features of Si_0.3_ and Si_0.5_ MEAs: (**a**,**b**) Si_0.3_ MEA, (**c**,**d**) Si_0.5_ MEA.

Figure 5 shows the morphologies of the fracture in the tensile-deformed samples of the Si_0.3_ and Si_0.5_ MEAs. There are numerous fine dimples in the Si_0.3_ sample, indicating excellent plastic stability and a ductile nature, as shown in Figure 5a,b. Some parallel bands (marked by yellow arrow in Figure 5b) can be seen inside the dimples, which are related to deformation twins (DTs), which is also a proof of superior ductility and strain hardening, as previously reported [8]. The Si_0.5_ MEA shows an obvious cleavage fracture, where river-like patterns and tearing ridges with several pits are clearly observed. Furthermore, many bumps with small and large sizes can be observed, indicating that the σ particles result in the reduction in plasticity.

**Figure 6:**
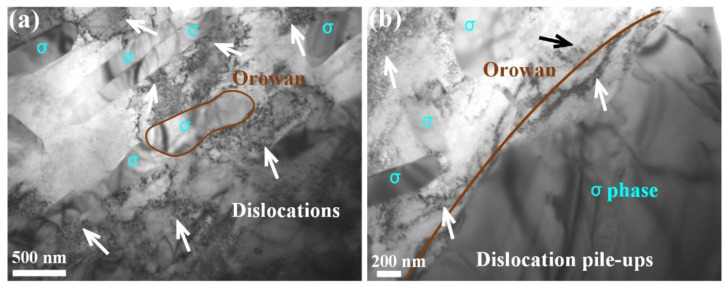
TEM images of post-fractural tensile deformation microstructures for the Si_0.5_ MEA.

In order to reveal the deformation hardening mechanism of the σ phase-contained Si_x_ MEA, the TEM microstructural characterization of the tensile-deformed samples was conducted. As exhibited in Figure 6a, abundant dislocations are distributed in the interspace of σ precipitation. No dislocation line is found within the σ particles, and numerous dislocations pile up and are bent around the precipitated particles, presenting an obvious Orowan hardening mechanism [23,24]. Figure 6b shows the thick dislocation pile-ups near the boundary of the large σ precipitation. It is the σ precipitation that strongly impedes the dislocation motion and promotes the high work hardening of the Si_x_ MEA (see Figure 4a). Furthermore, the σ precipitation can also increase the local stress and cause heterogeneous strain distribution [25], thereby resulting in the back stress strengthening and enhancing the strain hardening ability. As is reported in our previous study [24], the Orowan hardening mechanism is more effective than the shearing hardening mechanism in the enhancement of the strength and strain hardening capacity, so the Si_x_ MEA shows higher YS and a work hardening rate with the increase in the σ phase. However, because the σ phase is too hard to cut through, the plastic coordination during plastic deformation decreases and results in a reduction in plastic strain.

### 3.3. Wear Properties and Mechanisms

**Figure 7:**
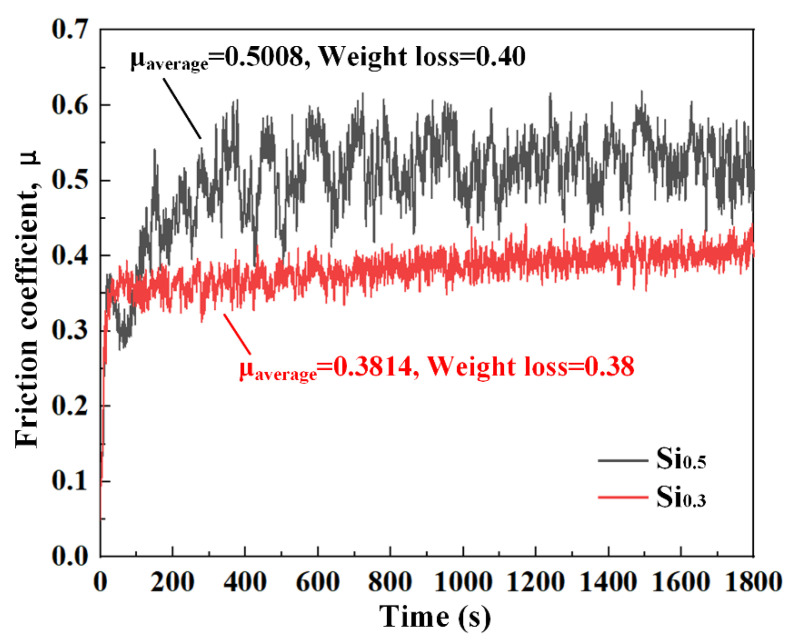
Friction coefficient of Si_0.3_ and Si_0.5_ MEAs during sliding against GCr15 steel balls from 20 N load.

In order to reveal the effect of Si addition on the wear properties and mechanisms of CrCoNi MEA, the typical Si_0.3_ (representing the single FCC phase structure) and Si_0.5_ (representing the FCC+ σ phase structure) MEAs are selected for the wear tests. Figure 7 displays the friction coefficient (μ) evolution histories of Si_0.3_ and Si_0.5_ MEAs during sliding under the load of 20 N. The friction coefficient of Si_0.3_ MEA shows a steady continuous rise with wear time, while that of Si_0.5_ MEA undergoes a decrease first and then an increase at the beginning running-in stage. This is because the temperature at the contacting surface increases as wear test progresses and aggravates the surface oxidation of the cladding layer, which plays a role in reducing the friction and thus making the friction coefficient decrease obviously [26]. When the oxide is worn out, the friction pair would continue to contact the deposited metal, and the friction coefficient would rise again and enter the stable friction stage. Furthermore, the friction coefficient of Si_0.5_ MEA exhibits a wider fluctuation amplitude than that of Si_0.3_ MEA throughout the wear process. This can be mainly observed with the σ precipitation in Si_0.5_ MEA, which leads to a large uneven distribution in the microstructure. The average friction coefficient of Si_0.3_ and Si_0.5_ MEAs are 0.3814 and 0.5008, respectively. The Si_0.5_ MEA shows an obviously higher friction coefficient than Si_0.3_ MEA, indicating that the increase in Si and the existence of the σ phase decrease the wear resistance of CrCoNiSi_x_ MEA. The wear loss of Si_0.5_ MEA is also a little lower than that of Si_0.3_ MEA, which goes against Archard’s rule [27]. According to Archard’s rule, the wear loss decreases with the increase in the hardness. Nevertheless, Archard’s rule is mainly based on a single wear mechanism, such as adhesion wear or abrasive wear [27,28]. Thus, this opposite trend is mainly related to the wear mechanism.

**Figure 8:**
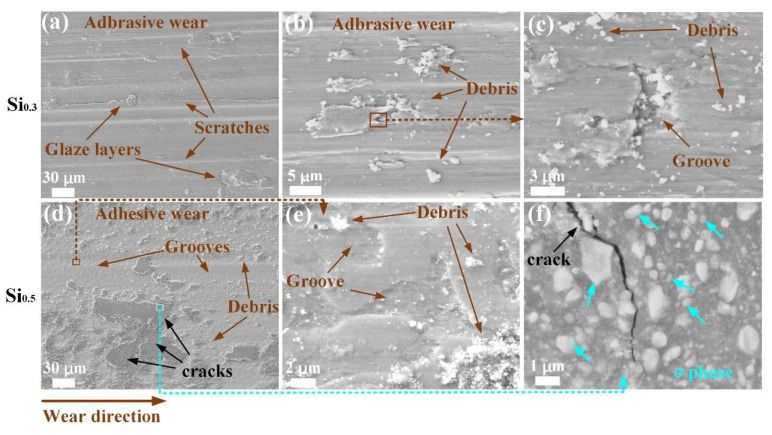
SEM images of wear surface of Si_0.3_ (**a**–**c**) and Si_0.5_ (**d**–**f**) MEAs, respectively. (**c**) is the amplified image taken from the brown rectangular area, (**e**) is the amplified image taken from the brown rectangular area, and (**f**) is the amplified image taken from the cyan rectangular area.

For revealing the wear mechanisms, the worn surfaces of Si_0.3_ and Si_0.5_ MEAs after sliding wear tests are given in Figure 8. From the worn surface morphology of Si_0.3_ MEA displayed in Figure 8a, numerous parallel scratches developed due to the ploughing of micro-asperities between two friction surfaces are presented along the wear direction, indicating a typical abrasive wear mechanism. In addition to the scratches, fine wear debris and discontinuous glaze layers also generate on the worn surface, as exhibited in Figure 8a,b. There are also small grooves near the debris, as shown in the magnified image (see Figure 8c) corresponding to the brown rectangular area in Figure 8b. The worn surface of Si_0.5_ MEA is nearly fully covered by the continuous compact glaze layers and a large amount of wear debris (Figure 8d), showing a typical adhesive wear mechanism. The glaze layers are believed to act as the protective interlayer to avoid the metal−metal direct contact during friction [26]. Additionally, an uneven surface in the interspace of glaze layers can be seen, which was revealed to be grooves through the corresponding magnified image (Figure 8e), indicating that there are abundant small grooves in the surface of Si_0.5_ MEA. Moreover, there are many micro-cracks in the glaze layers, as marked by the black arrows in Figure 8d. These cracks were reportedly induced due to the stick-slip behavior and high stress localizations during repetitive sliding, and they would result in the failures of glaze layers or oxide patches after their growth to a critical thickness [29,30,31]. Figure 8f gives us the magnified image of the crack region, from which large and small sized σ particles are visible in the glaze layer, and the crack expands along the σ particle boundary. It can be drawn that the hard σ phase contributes to the formation of the crack. These inhomogeneous distributions of the wear debris, glaze layers, together with the σ particles, contribute to the significant fluctuations of friction coefficients, as displayed in Figure 7.

In a word, with the increase in Si content, the main wear mechanisms change from plastic deformation and abrasion wear for the σ-free Si_0.3_ MEA to adhesion, delamination, and mild oxidation wear for the σ phase-contained Si_0.5_ MEA. During the wear process, the σ particles may be ribbed out by the GCr15 counterpart and stay on the friction surface, which may lead to a three-body-wear form [32]. The σ particles could dig into the FCC matrix and plow out the alloy, which is probably related to the abundant grooves in Si_0.5_ MEA. The wear loss is partly caused by the debonding of σ particles and the hard σ phase friction. Therefore, the Si_0.5_ MEA exhibits more wear loss and a lower wear resistance than the Si_0.3_ MEA.

## 4. Conclusions

In this study, the microstructure evolution, mechanical, wear properties, and mechanisms involved with increasing the Si content in CrCoNi MEA were systematically investigated. The obtained conclusions are summarized as follows:

(1) The CrCoNiSi_x_ MEA keeps the single FCC phase when x is less than 0.3, while the σ phase appears when x = 0.4 and increases with increasing the Si content, exhibiting a transition from a uniform distribution of small-sized σ precipitates to a large- and-small-particle distributed network structure and, eventually, to an FCC phase distributed in the σ-phase matrix structure.
(2) The tensile and compressive yield strengths are enhanced with the increase in Si content, which is attributed to the Orowan hardening mechanism from the σ phase. The increased σ phase also effectively improves the hardness of CrCoNiSi_x_ MEA at both room and high temperature, demonstrating the effective enhancement of the σ phase in hardness at high temperatures.
(3) The σ phase-contained Si_x_ MEA shows a lower wear resistance than the σ-free Si_x_ MEA due to the transformation of the main wear mechanism from abrasion wear to adhesion wear.
(4) Small Si addition promotes solution strengthening and synergistically enhances the strength and plasticity; more content of Si contributes to precipitation strengthening of the σ phase, thus effectively enhancing the strength and hardness but with a reduction in plasticity.

## Data Availability

The data that support the findings of this study are available from the corresponding authors upon reasonable request.
